# *Streptomyces* in biotechnology: An anotated selection of world wide web sites relevant to the topics in *microbial biotechnology*

**DOI:** 10.1111/1751-7915.13677

**Published:** 2020-10-04

**Authors:** Lawrence P. Wackett

**Affiliations:** ^1^ Department of Biochemistry, Molecular Biology & Biophysics BioTechnology Institute University of Minnesota St Paul MN 55108 USA

## *Streptomyces* protein excretion

https://academic.oup.com/femsle/article/365/22/fny250/5123710

This review discusses Gram‐positive bacterial protein secretion systems, with a particular emphasis on the genus *Streptomyces*.

## Metabolic engineering of *Streptomyces*

https://onlinelibrary.wiley.com/doi/abs/10.1002/biot.201700465

Because of the propensity for *Streptomyces* making useful secondary metabolites, metabolic engineering of this genus is particularly interesting to many. This review article focuses on that topic.

## *Streptomyces* plant growth stimulation

https://link.springer.com/article/10.1007/s00253-018-09577-y

This mini‐review focuses on the ability of *Streptomyces* strains to inhabit soil near plants and to stimulate their health and growth.

## *Streptomyces lividans*

https://microbewiki.kenyon.edu/index.php/Streptomyces_lividans

This page discusses not only *S. lividans* but also *Streptomyces* in general. *S. lividans is* used industrially as a protein secretion host.

## DSMZ *Streptomyces* strains

https://bacdive.dsmz.de/search?search=streptomyces&submit=

This strain collection website contains information on thousands of *Streptomyces* strains that can be obtained from the collection.

## *Streptomyces* taxonomic list

https://www.ncbi.nlm.nih.gov/Taxonomy/Browser/wwwtax.cgi

This NCBI site lists well‐studied *Streptomyces* strains, as well as isolates that have not been studied in detail.

## *Streptomyces* annotation server

http://strepdb.streptomyces.org.uk/cgi-bin/dc3.pl?accession=AL645882&start=4291472&end=4302043&iorm=map&width=900

This database was made to share resources for those studying Actinobacteria, with a focus on *Streptomyces*.

## *Streptomyces* phage database

https://phagesdb.org/hosts/genera/4/

This database focuses on bacteriophages infecting *Streptomyces* species.

## Thirty new *Streptomyces* genomes

https://www.nature.com/articles/s41597-020-0395-9

This recent paper describes the sequencing of thirty new *Streptomyces* genomes and their mining for novel biosynthetic gene clusters.

## *Streptomyces coelicolor* KEGG

https://www.genome.jp/kegg-bin/show_organism?org=sco

*Streptomyces coelicolor* is a model *Streptomyces* species, and this database page links to the genome, metabolic networks and enzymes of the organism.

## *Streptomyces:* Microbiology Society

https://www.microbiologyresearch.org/content/streptomyces

This scientific society page provides a good overview of *Streptomyces* bacteria.

https://www.sciencedirect.com/science/article/pii/S0944501314000573

This study describes a protocol and results from the use of Matrix‐Assisted Laser Desorption/Ionization Time‐Of‐Flight Mass Spectrometry (MALDI‐TOF‐MS) for identifying *Streptomyces* isolates.

## *Streptomyces coelicolor* strain A3(2): BioCyc

https://biocyc.org/SCO/organism-summary?object=SCO

This database page contains information on the metabolism and enzymes of the organism.

## NCBI *Streptomyces* genomes

https://www.ncbi.nlm.nih.gov/genome/?term=streptomyces

This database page provides links to information on *Streptomyces* genomes. At this time, there are 3740 genome assemblies.

## *Streptomyces* genome diversity

https://mbio.asm.org/content/10/5/e01533-19

Insertions and deletions occur commonly in *Streptomyces*, leading to a large diversity of genomes over time.

## Genome mining in *Streptomyces*

https://www.sciencedirect.com/science/article/pii/S2001037020303135

This mini‐review discusses approaches for the identification for secondary metabolite biosynthetic gene cluster in *Streptomyces* species.

## Bioinformatics of *Streptomyces*

https://scholars.unh.edu/cgi/viewcontent.cgi?article=2307&context=thesis

This master’s thesis describes approaches for conducting bioinformatics analyses of *Streptomyces* genomes.

## *Streptomyces* biosynthetic gene clusters

https://mibig.secondarymetabolites.org/query

This database contains information on biosynthetic gene clusters broadly. *Streptomyces* and their products are prominently featured in the database.

